# Incidence and prevalence of gout in Eastern China from 2011 to 2021: a retrospective population-based study

**DOI:** 10.1080/07853890.2025.2561230

**Published:** 2025-09-19

**Authors:** Ke Liu, Ding Ye, Hao Lin, Yexiang Sun, Peng Shen, Jianbing Wang, Zhiqin Jiang, Yingying Mao, Kun Chen

**Affiliations:** ^a^ Department of Epidemiology, School of Public Health, Zhejiang Chinese Medical University, Hangzhou, China; ^b^ Department of Chronic Disease and Health Promotion, Yinzhou District Center for Disease Control and Prevention, Ningbo, China; ^c^ Department of Public Health, and Department of Endocrinology of the Children’s Hospital, Zhejiang University School of Medicine, Hangzhou, China; ^d^ Department of Public Health, Second Affiliated Hospital, Zhejiang University School of Medicine, Hangzhou, China

**Keywords:** Epidemiology, gout, incidence, prevalence, trend

## Abstract

**Background:**

To characterize the burden of gout by estimating the temporal trends in prevalence and incidence, and assessing age- and gender-specific patterns in Yinzhou, Ningbo, China (2011–2021).

**Methods:**

A population-based retrospective study was conducted using the Yinzhou Regional Health Information Platform. Poisson regression estimated the 95% confidence intervals (CIs) for prevalence and incidence rates, and relative risks for both rates across subgroups. Age-standardized incidence (ASIR) and prevalence rates (ASPR) were calculated based on China’s 2020 census. The average annual percent changes (AAPCs) of standardized rates were calculated to estimate the secular trends using joinpoint regression analysis.

**Results:**

Of the over 1.1 million resident adults, 23,967 gout cases were identified over an eleven-year period. The total incidence and prevalence rates from 2011 to 2021 were 211.29 (95% CI: 208.28–214.33)/100,000 person-years and 2.16% (95% CI: 2.13% to 2.19%), respectively. The incidence and prevalence rates of gout was more than twice as heavy in men than women, and exhibited a rising tendency with advancing age, particularly for elderly men. Additionally, lower education attainment, obesity, and unfavorable lifestyle contributed significantly to the burden of gout. ASIR (AAPC: 5.37, 95% CI: 0.30–10.70) and ASPR (AAPC: 7.75, 95% CI: 6.29–9.23) increased significantly over the 11-year period, though ASIR in women remained stable.

**Conclusions:**

The incidence and prevalence of gout increased over time, showing age- and gender-specific patterns. The healthcare authorities need to focus on the burden of gout and guide targeted prevention and treatment strategies for gout.

## 1. Introduction

Gout is a common inflammatory arthritis and occurs when sustained elevation of serum urate levels (hyperuricaemia) leads to the formation and deposition of monosodium urate crystals in and around the joints [1]. Untreated gout increased the risk of destructive arthritis, cardiovascular events, and long-term comorbidity, placing a substantial burden on the global health system [2–6]. To be noted, the incidence and prevalence of gout have been on the rise for the past 30 years, with the age-standardized incidence of 109.07 and prevalence of 653.82 per 100,000 worldwide in 2021, which is projected to continue to increase in the future [7–9]. There is also an issue should not be overlooked that the burden of gout exhibited considerable regional and ethnic heterogeneity [10], with the highest prevalence in Oceania, North America, and Western Europe, among which the highest point prevalence estimates are for high-income countries in North America [7,11,12].

To date, limited and inconsistent epidemiological burden studies of gout has been collected from China [13]. For example, previous studies from Taiwan reported the highest prevalence of gout at 11.7% and found that a higher prevalence in urban areas (0.67%) compared with that reported in rural areas (0.16%) [14,15]. On a larger scale, a previous meta-analysis systematically collected 44 epidemiological surveys covering 16 provinces, demonstrating that the prevalence rates of gout during 2000–2014 was about 1.1% in China [16]. Subsequently, an updated study encompassing 31 provinces of mainland China reported a standardized prevalence of 3.2%, manifesting significant variation by geographic regions, with the highest rate of 9.9% in Tibet [17]. However, to date, studies on the epidemiological characteristics of gout in China are mainly based on traditional epidemiological surveys, covering a small range of populations. Moreover, a majority of studies reported the prevalence rate rather than incidence rate, thus there is a lack of large-sample studies related to the trend of incidence.

Routinely collected electronic healthcare records are valuable for examining the burden of chronic diseases, because of the availability of data on a large number of patients that are broadly representative of the population, which is of importance to inform policy making with respect to gout management in China. Therefore, this study used data on the Yinzhou Regional Health Information Platform (YRHIP) from 2011 to 2021 to quantify the incidence and prevalence rates of gout in Yinzhou district, Ningbo city, China, and to investigate their patterns across sex, age groups and targeted modifiable risk factors.

## 2. Methods

### 2.1. Data sources

This population-based study was performed using the YRHIP database, which incorporated three main data source including hospital electronic medical records, public health management data, and community health management data. Initially, YRHIP was constructed by Yinzhou District Health Bureau in 2005 and essentially completed in 2010. YRHIP connects to three general hospitals, 24 community health service centers and 287 community service stations in the region, covering district hospitals and affiliated community health service centers and township health centers in the whole region. As of 2016, the coverage rate of Yinzhou’s household population reached 98%, basically realizing full coverage of all health-related data of local residents from birth to death. This study was approved by the Zhejiang Chinese Medical University Ethics Committee (Institutional Review Board no. 20231208-2), which waived the requirement for informed consent in accordance with national regulations and institutional policies. All data were safeguarded in a secure local server environment, accessible only to designated staff, in accordance with data protection principles. All methods were carried out in accordance with the Declaration of Helsinki.

### 2.2. Study design and population

Data covering 1st January 2011 to 31st December 2021 was used in this study. Eligibility for inclusion in this study population is listed as follows: (i) regular residents of Yinzhou District with a valid identification number from 1st January 2011 to 31st December 2021, (ii) recorded in YRHIP for more than one year during the survey period, and (iii) aged over 18 and less than 110 years. The observation period commenced on 1st January 2011 or the date of first record on the YRHIP plus one year. The observation period terminated on the earliest of (i) 31st December 2021, (ii) the date of moving out of Yinzhou (iii) the date of death, or (iv) the date of diagnosing gout.

### 2.3. Case identification

Patients with gout was identified using the International Classification of Diseases-10 code (M10) or diagnostic text (Table S1). Natural language processing and rheumatologists was applied to standardize the unstructured diagnostic text. Incident diagnoses were those patients with no gout-related diagnosis within 1 year of registration and a first diagnosis of gout one year later, with gout as the primary diagnosis. The 1-year wash-out period was imposed to minimize the risk of prevalent cases being identified as incident ones. Prevalent diagnoses were those with a record of gout at any point before the end of the observation period. Early onset gout was defined as individuals who are diagnosed with gout before the age of 40.

If the diagnostic text contained words such as ‘undetermined’, ‘uncertainty’, ‘?’ and other synonyms, the patients were considered as uncertain gout, which was excluded in the primary analysis but included in the sensitivity analysis.

### 2.4. Statistical analysis

Summary statistics are presented as frequencies and percentages for categorical variables and mean, standard deviation (SD) for continuous variables. Student’s t-test for continuous variables and the chi-square (*χ^2^*) test for categorical variables were used to compare male and female differences. The incidence rates per 100,000 person-years and prevalence rates per 100 people overall and by age (18–29, 30–39, 40–49, 50–59, 60–69, 70–79 and ≥ 80 years), sex (male and female), body mass index [BMI] (normal [18.5–23.9 kg/m^2^], underweight [<18.5 kg/m^2^], overweight [24–27.9 kg/m^2^] and obesity [≥28 kg/m^2^]), ethnicity (Han Chinese, and non-Han Chinese), education levels (college and above, high/technical/secondary school, lower secondary/primary school and other), current smoking (yes and no) and current drinking (yes and no) were calculated. Missing data on covariates were coded as a missing indicator and presented as a separate category. Poisson regression was used to calculate 95% confidence intervals (CIs) of incidence and prevalence, and relative risks for both rates across subgroups.

Furthermore, we calculated annual age-standardized rates (ASRs) based on the 2020 Chinese national census population data, and the average annual percentage changes (AAPC) of ASR of incidence and prevalence were employed to estimate the temporal trends in burden of gout from 2011 to 2021 by joinpoint model. To fit the optimal joinpoint model, we applied Joinpoint Regression Analysis software program to start with the minimum number of joinpoint and test whether more joinpoints are statistically significant and should be added to the model [18]. The AAPC can use the segmented annual percent changes to summarise and compare the rates of change over time and identify long-term trends, even if they are unstable [18]. When the AAPC value and its lower 95% CI are positive, the ASR exhibits an increasing trend. Conversely, a descending trend of ASR is observed when the AAPC value and its upper 95% CI is negative. Others represent that ASR is stable over time.

Sensitivity analyses were conducted to assess the robustness of the results: (i) extending the wash-out period for new cases to two years, (ii) including uncertain gout patients, (iii) standardizing rates based on the global standard population 2021, (iv) excluding all out- and in-migrating populations to eliminate the effect of administrative realignment on study results and (v) including cases identified by gout-specific medication dispensation (colchicine and urate-lowering therapies) (Table S2).

All statistical analyses were conducted with R (version 4.3.0) and Joinpoint (version 4.9.1.0), and a two-sided *P* value < 0.05 was considered as statistically significant.

## 3. Results

**Figure 1.**
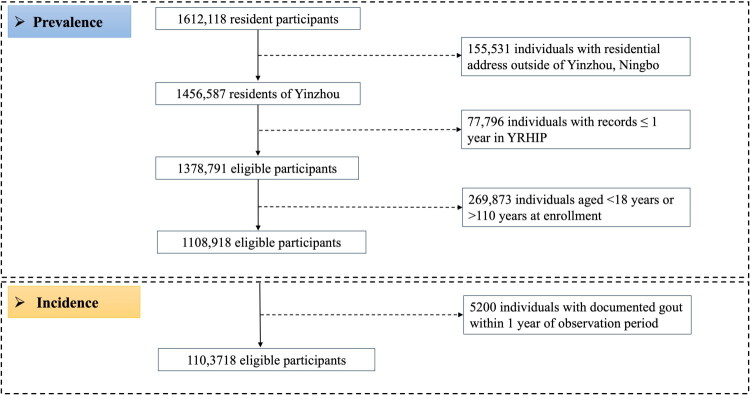
Flow chart of study participants.

A total of 1108,918 individuals aged over 18 years in Yinzhou District were included, of which 533,370 were male and 575,548 were female, with a sex ratio of 1:1.08 (Figure 1; Table 1). The mean age of the participants was 43.73 (SD = 16.14) years; only approximately 18.11% had a college education or above; the majority were Han-Chinese (97.80%) and embraced a favorable lifestyle, of which 78.83% were nonsmokers, 65.34% were nondrinkers and 63.95% maintained a normal BMI (Table 1).

**Table 1.** Characteristics of the study participants (≥18 years) in YRHIP.

Characteristics	Total	Male	Female	*P* value
	(*N* = 1108,918)	(*N* = 533,370)	(*N* = 575,548)	
**Age [year, Mean (SD)]**	43.73 (16.14)	44.18 (15.95)	43.32 (16.31)	<0.001
**Age groups, N (%)**				
18∼	252873 (22.80)	112729 (21.14)	140144 (24.35)	
30∼	261539 (23.59)	124567 (23.35)	136972 (23.80)	
40∼	237190 (21.39)	118507 (22.22)	118683 (20.62)	
50∼	172134 (15.52)	85998 (16.12)	86136 (14.97)	
60∼	101963 (9.19)	51898 (9.73)	50065 (8.70)	
70∼	53556 (4.83)	26907 (5.04)	26649 (4.63)	
≥80	29663 (2.67)	12764 (2.39)	16899 (2.94)	
**Nation, *N* (%)**				<0.001
Han-Chinese	1084534 (97.80)	524724 (98.38)	559810 (97.27)	
Other	4103 (0.37)	1991 (0.37)	2112 (0.37)	
Missing	20281 (1.83)	6655 (1.25)	13626 (2.37)	
**Education level, *N* (%)**				
College and above	200857 (18.11)	96184 (18.03)	104673 (18.19)	
High/Technical/technical secondary school	171088 (15.43)	88499 (16.59)	82589 (14.35)	
Lower secondary/primary school	530237 (47.82)	255468 (47.90)	274769 (47.74)	
Other	191997 (17.31)	87964 (16.49)	104033 (18.08)	
Missing	14739 (1.33)	5255 (0.99)	9484 (1.65)	
**Current smoking, *N* (%)**				
No	874116 (78.83)	356159 (66.78)	517957 (89.99)	
Yes	130428 (11.76)	127994 (24.00)	2434 (0.42)	
Missing	104374 (9.41)	49217 (9.23)	55157 (9.58)	
**Current drinking, *N* (%)**				
No	724598 (65.34)	297408 (55.76)	427190 (74.22)	
Yes	279794 (25.23)	186604 (34.99)	93190 (16.19)	
Missing	104526 (9.43)	49358 (9.25)	55168 (9.59)	
**Body mass index, *N* (%)**				
Normal	709173 (63.95)	333575 (62.54)	375598 (65.26)	
Low mass index	42976 (3.88)	12489 (2.34)	30487 (5.30)	
Overweight	227646 (20.53)	124771 (23.39)	102875 (17.87)	
Obesity	38133 (3.44)	18959 (3.55)	19174 (3.33)	
Missing	90990 (8.21)	43576 (8.17)	47414 (8.24)	

### 3.1. Incidence

After excluding 5,200 individuals with a gout diagnosis within the one-year washout period, a total of 18,767 (13,435 men [71.59%], 5,332 women [28.41%]) newly incident gout cases were identified during the observation period (Figure 1), with the mean age of onset of 60.47 (SD = 15.81) years. The total follow-up person-years amounted to 8882171.28 years, and the mean follow-up time was 8.05 (SD = 3.30) years (Table 2). The incidence rates of gout and early-onset gout from 2011 to 2021 were 211.29 (95% CI: 208.28 to 214.33)/100,000 person-years and were 73.27 (95% CI: 70.05–76.61)/100,000 person-years, respectively. The incidence in males was 313.08 (95% CI: 307.81–318.42)/100,000 person-years, and (116.14, 95% CI: 113.04–119.3)/100,000 person-years in females, with crude incidence rate ratio of 2.70 (95% CI: 2.61–2.78, *p* < 0.001) (Table 2). In 2021, the total population incidence rate increased with age, reaching a peak in those with aged 70–80 years with 769.97 (95% CI: 712.68–830.64)/100,000 person-years, and sex-specific trends across age groups were consistent with the total population (Table 3). Additionally, increased BMI, lower education levels and unfavorable lifestyle significantly increase the risk of incident gout, with crude incidence rate ratios ranging from 1.27 for secondary education to 2.61 for obesity (Table 2).

**Table 2.** Incidence rates (with 95% CI) of gout per 100,000 person-years of adults (≥18 years) during 2011–2021.

Characteristics	Events	Person years	Incidence rate (/100,000 person-years, 95% CI)	Crude rate ratio	*P value*
**Total**	18767	8882171.28	211.29 (208.28–214.33)	–	–
**Sex**					
Female	5332	4591002.81	116.14 (113.04–119.30)	1.0 (ref)	<0.001
Male	13435	4291168.47	313.08 (307.81–318.42)	2.70 (2.61–2.78)	
**Nation**					
Han-Chinese	18651	8693623.46	214.54 (211.47–217.64)	1.0 (ref)	
Other	28	27924.08	100.27 (66.63–144.92)	0.47 (0.32–0.68)	<0.001
Missing	88	160623.74	–	–	–
**Education level**					
College and above	1710	1408624.46	121.40 (115.71–127.29)	1.0 (ref)	
High/Technical/technical secondary school	2150	1396702.59	153.93 (147.50–160.58)	1.27 (1.19–1.35)	<0.001
Lower secondary/primary school	12296	4369724.12	281.39 (276.44–286.41)	2.32 (2.20–2.44)	<0.001
Other	2548	1603414.78	158.91 (152.80–165.20)	1.31 (1.23–1.39)	<0.001
Missing	63	103705.33	–	–	–
**Current smoking**					
No	14025	6946531.43	201.90 (198.57–205.27)	1.0 (ref)	
Yes	3832	1040853.55	368.16 (356.59–380.00)	1.82 (1.76–1.89)	<0.001
Missing	910	894786.30	–	–	–
**Current drinking**					
No	11500	5610578.96	204.97 (201.24–208.75)	1.0 (ref)	
Yes	6346	2375726.94	267.12 (260.59–273.77)	1.30 (1.26–1.34)	<0.001
Missing	921	895865.38	–	–	–
**Body mass index**					
Normal	10211	5597069.76	182.43 (178.91–186.01)	1.0 (ref)	
Low mass index	551	340600.98	161.77 (148.55–175.86)	0.89 (0.81–0.97)	<0.001
Overweight	5985	1844984.75	324.39 (316.23–332.72)	1.78 (1.72–1.84)	0.006
Obesity	1528	320447.58	476.83 (453.22–501.36)	2.61 (2.48–2.76)	<0.001
Missing	492	779068.21	–	–	–

**Table 3.** Incidence (/100,000 person-years, 95% CI) and prevalence (/100 people, 95% CI) of gout by age group and sex in 2021.

Age groups	Total		Male		Female	
	Incidence rate	Prevalence rate	Incidence rate	Prevalence rate	Incidence rate	Prevalence rate
18∼	119.57 (76.61–177.90)	3.02 (2.40–3.75)	288.37 (182.80–432.70)	6.38 (4.97–8.05)	8.27 (0.21–46.06)	0.74 (0.38–12.95)
30∼	200.42 (180.79–221.59)	6.81 (6.47–7.16)	413.25 (371.37–458.55)	125.24 (118.43–132.33)	24.95 (16.30–36.56)	19.50 (17.07–22.17)
40∼	285.18 (260.53–311.53)	121.24 (116.44–126.19)	519.30 (471.43–570.72)	199.20 (190.36–208.34)	68.14 (52.12–87.52)	48.08 (43.92–52.53)
50∼	422.05 (393.43–452.20)	201.06 (195.26–206.99)	660.32 (609.64–714.09)	295.14 (285.18–305.36)	187.00 (160.68–216.41)	108.17 (102.20–114.38)
60∼	606.03 (564.79–649.50)	292.31 (283.92–300.87)	936.80 (864.15–1013.92)	418.27 (404.12–432.79)	280.66 (241.88–323.90)	166.01 (157.13–175.27)
70∼	769.97 (712.68–830.64)	402.31 (390.21–414.69)	1098.29 (1000.83–1202.69)	570.70 (550.29–591.68)	455.70 (395.02–523.06)	236.10 (223.12–249.64)
≥80	559.58 (502.24–621.66)	525.61 (509.49–542.11)	818.79 (718.26–929.44)	710.98 (683.90–738.86)	329.05 (269.93–397.28)	356.18 (337.92–375.17)

**Figure 2.**
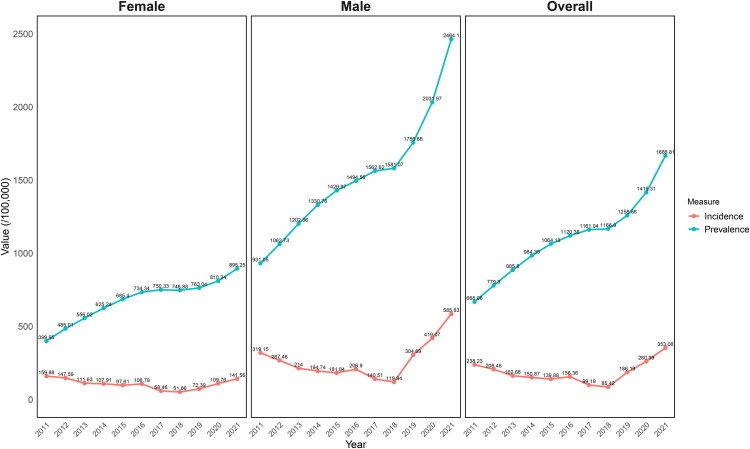
Total and sex-specific annual incidence and prevalence of gout in Yinzhou District, Ningbo, China, 2011–2021.

Normalized to China’s 2020 census data, the age-standardized incidence rates (ASIR) continued to decline from 238.23 (95% CI: 225.17–251.30)/100,000 person-years in 2011 to a nadir of 85.42 (95% CI: 79.14–91.70)/100,000 person-years in 2018, and then maintained an upward trend to 353.08 (95% CI: 339.11–367.06)/100,000 person-years in 2021. This pattern mirrored the pronounced drop and rebound observed in both sexes (Table 4; Figure 2). In terms of secular trends, differently, increasing trends of age-standardized incidence rate over the 11-year period were observed in the overall population (AAPC: 5.37, 95% CI: 0.30–10.70, *p* = 0.038) and in males (AAPC: 8.56, 95% CI: 3.53–13.82, *p* < 0.001), while nonsignificant change was observed in females (AAPC: −1.59, 95% CI: −7.97–5.23, *p* = 0.639). For early-onset gout, increasing trends of ASIR was detected only among males (AAPC: 14.69, 95% CI: 4.94–25.36, *p* = 0.003), but not in the total population (AAPC: 7.71, 95% CI: −1.47–17.76, *p* = 0.102) or in females (AAPC: −9.04, 95% CI: −19.18–2.37, *p* = 0.116) (Table S2).

**Table 4.** Annual age-standardized incidence (/100,000 person-years, 95% CI) and prevalence (/100 people, 95% CI) of gout by sex.

Calendar year	Total		Male		Female	
	Incidence rate	Prevalence rate	Incidence rate	Prevalence rate	Incidence rate	Prevalence rate
2011	238.23 (225.17–251.30)	0.67 (0.65–0.69)	319.15 (297.57–340.72)	0.93 (0.90–0.96)	159.88 (144.80–174.97)	0.40 (0.38–0.42)
2012	206.46 (195.52–217.41)	0.78 (0.76–0.80)	267.46 (249.70–285.22)	1.06 (1.03–1.09)	147.59 (134.50–160.68)	0.49 (0.46–0.51)
2013	162.66 (153.94–171.39)	0.89 (0.87–0.91)	214.00 (199.87–228.13)	1.20 (1.17–1.23)	111.63 (101.33–121.92)	0.56 (0.53–0.58)
2014	150.87 (142.92–158.83)	0.98 (0.96–1.00)	194.74 (181.98–207.51)	1.33 (1.90–1.36)	107.91 (98.32–117.50)	0.63 (0.60–0.65)
2015	139.88 (132.40–147.35)	1.06 (1.04–1.08)	181.84 (169.77–193.92)	1.43 (1.40–1.46)	97.61 (88.81–106.42)	0.69 (0.66–0.71)
2016	156.36 (148.23–164.49)	1.12 (1.10–1.14)	206.90 (193.57–220.22)	1.50 (1.46–1.53)	106.79 (97.31–116.27)	0.73 (0.71–0.76)
2017	99.19 (92.55–105.82)	1.16 (1.14–1.18)	140.51 (129.23–151.78)	1.56 (1.53–1.60)	58.46 (51.27–65.65)	0.75 (0.73–0.77)
2018	85.42 (79.14–91.70)	1.17 (1.15–1.19)	119.94 (109.12–130.76)	1.58 (1.55–1.62)	51.86 (45.13–58.58)	0.75 (0.72–0.77)
2019	186.19 (176.79–195.60)	1.26 (1.24–1.28)	304.69 (287.12–322.26)	1.76 (1.72–1.79)	72.39 (64.38–80.40)	0.76 (0.74–0.79)
2020	260.99 (250.00–271.97)	1.42 (1.39–1.44)	419.07 (398.27–439.88)	2.03 (1.99–2.07)	109.76 (100.34–119.19)	0.81 (0.79–0.83)
2021	353.08 (339.11–367.06)	1.67 (1.64–1.69)	585.83 (556.14–615.51)	2.46 (2.42–2.51)	141.56 (131.12–152.01)	0.90 (0.87–0.92)

### 3.2. Prevalence

Of the 1,108,918 study participants, 23,967 (17,275 men [72.08%], 6,692 women [27.92%]) were diagnosed as having had a gout during 2011–2021. The prevalence of gout was 2.16% (95% CI: 2.13% to 2.19%) over the 11-year period, with a 2.79-fold higher prevalence in males (3.24%, 95% CI: 3.19% to 3.29%) compared to females (1.16%, 95% CI: 1.14% to 1.19%) (Table 5). The trends of prevalence with age in males and females were similar: the prevalence continues to increase and peaks after the age of 80 in 2021 (Table 3). Also, prevalence varied by education levels, BMI and lifestyle factors (Table 5).

**Table 5.** Prevalence rates (with 95% CI) of gout per 100 people of adults (≥18 years) during 2011–2021.

Characteristics	Events	Eligible population	Prevalence rate (/100 people, 95% CI)	Crude rate ratio	*P value*
**Total**	23967	1108918	2.16 (2.13–2.19)	–	–
**Sex**					
Male	17275	533370	3.24 (3.19–3.29)	1.0 (ref)	
Female	6692	575548	1.16 (1.14–1.19)	2.79 (2.71–2.87)	<0.001
**Nation**					
Han-Chinese	23812	1084534	2.20 (2.17–2.22)	1.0 (ref)	
Other	30	4103	0.73 (0.49–1.04)	0.28 (0.24–0.33)	<0.001
Missing	125	20281	–	–	–
**Education level**					
College and above	2239	200857	1.11 (1.07–1.16)	1.0 (ref)	
High/technical/technical secondary school	2729	171088	1.60 (1.54–1.66)	1.43 (1.35–1.51)	<0.001
Lower secondary/primary school	15500	530237	2.92 (2.88–2.97)	2.62 (2.51–2.74)	<0.001
Other	3418	191997	1.78 (1.72–1.84)	1.60 (1.51–1.68)	<0.001
Missing	81	14739	–	–	–
**Current smoking**					
No	18018	874116	2.06 (2.03–2.09)	1.0 (ref)	
Yes	4863	130428	3.73 (3.62–3.83)	1.81 (1.75–1.87)	<0.001
Missing	1086	104374	–	–	–
**Current drinking**					
No	14881	724598	2.05 (2.02–2.09)	1.0 (ref)	
Yes	7986	279794	2.85 (2.79–2.92)	1.39 (1.35–1.43)	<0.001
Missing	1100	104526	–	–	–
**Body mass index**					
Normal	13019	709173	1.84 (1.80–1.87)	1.0 (ref)	
Low mass index	705	42976	1.64 (1.52–1.77)	0.89 (0.83–0.96)	<0.001
Overweight	7653	227646	3.36 (3.29–3.44)	1.83 (1.78–1.88)	0.004
Obesity	1913	38133	5.02 (4.79–5.25)	2.73 (2.60–2.87)	<0.001
Missing	677	90990	–	–	–

From 2011 to 2021, the annual age-standardized prevalence rates (ASPR) ranged from 0.67% (95% CI: 0.65% to 0.69%) in 2011 to 1.67% (95% CI: 1.64% to 1.69%) in 2021, manifesting an overall continuing upward trend (AAPC: 7.75, 95% CI: 6.29–9.23, *p* < 0.001) (Table 4; Figure 2). Similarly, there was a significant rise in age-standardized prevalence over the 11-year period both in male (AAPC: 8.95, 95% CI: 6.05–11.9%, *p* < 0.001) and female (AAPC: 7.81, 95% CI: 5.43–10.24, *p* < 0.001)

### 3.3. Sensitivity analysis

In the sensitivity analysis, the results obtained by including all uncertain gout cases were comparable to the main estimates (212.49, 95% CI: 209.46–215.52/100,000 person-years for incidence; 2.18%, 95% CI: 2.15% to 2.20% for prevalence), and incorporating medication-based gout diagnoses led to increased estimates, with an incidence of 225.27 (95% CI: 222.16–228.42)/100,000 person-years and a prevalence of 2.30% (95% CI: 2.28% to 2.33%). In contrast, both rates decreased when excluding all out- and in-migrating participants (180.59, 95% CI: 177.66–183.52/100,000 person-years for incidence; 2.01%, 95% CI: 1.98% to 2.04% for prevalence). Standardized to the global 2021 Standardized Population, both ASIR 413.65 (95% CI: 399.81–427.48)/100,000 person-years and ASPR 2.15% (95% CI: 2.12% to 2.18%) increased in 2021, compared to the 2020 Chinese national census population. Furthermore, when extending the wash-out period for new cases to 2 years, the lower bound of the incidence was observed (188.42, 95%CI: 185.57**–**191.28)/100,000 person-years.

## 4. Discussion

Based on the data from the Yinzhou Regional Health Information Platform, our retrospective study found that the total incidence of gout remained stable from 2011 to 2021, despite an upward trend since 2018, and the prevalence has continued to increase, affecting 2.2% of adults in 2021. The burden of gout exhibits considerable sex and age disparities, with the elderly suffering the heaviest burden of gout, and men accounting for more than twice the burden of gout as women. Furthermore, increased BMI, lower education levels and unfavorable lifestyle significantly increase the burden of gout.

In terms of the distribution of the population, the prevalence of gout in the Yinzhou District is significantly lower than the 3.2% reported in a previous study synthesizing data from 31 provinces in China [17]. Notably, in this nationwide survey of the general Chinese adult population, there are large differences in the prevalence of gout between provinces, with the prevalence in the top ten regions ranging from nearly 10% in Tibet to approximately 6% in Szechwan, excluding Zhejiang Province, where the Yinzhou District is located. Reviewing more recent studies, gout prevalence in our study also appears to be significantly lower when comparing populations outside China. Yokose C and colleagues recently reported a gout prevalence of 5.1% using the National Health and Nutrition Examination Survey data from 2017 to 2018 [19]. A systematic review of 12 studies in the Australian population across ages showed that self-reported diagnosis of gout ranged from 4.5% to 6.8%, while estimated prevalence ranging between 1.5% and 2.9% using electronic coding data from general practitioners or wastewater estimates of allopurinol consumption [20]. Nevertheless, EPISER2016 conducted a population-based study to estimate the prevalence of gout in Spain at 2.4% [21], which is close to our results. In comparison, fewer and varied research addressed the incidence than the prevalence of gout. For instance, Helget et al. estimated that the incidence of gout per 1,000 patient-years over a 10-year period increased from 5.8 cases in 2005 to 7.4 cases in 2014 using data from the U.S. National Veterans Health Administration (VHA) [22]. Another study utilized a nationally representative sample of the general population sample in the UK, and reported an increase in the incidence of gout from 2.13 in 1990 to 2.54 per 1,000 person-years in 2019 [23]. Some of the differences across studies reflect differences in the study design or case ascertainment, while others could signify differences in genetic susceptibility [24], lifestyle and comorbid risk factor profiles among the different geographical populations and time periods, all of which may confer different risks of gout, highlighting the need for accurate regional and national data. Notably, the marked increase in the incidence of early-onset gout, particularly among young males, highlights the importance of early identification and targeted prevention strategies for younger individuals at risk with characteristics such as high BMI, unhealthy lifestyle behaviors, and ­dyslipidemia [25].

The incidence and prevalence of gout increase with advancing age, with a pattern that is seen over the entire lifespan in both sexes. Interpretably, aging can cause renal morphologic and pathophysiologic dysfunction, resulting in impaired uric acid excretion and elevated serum levels. Moreover, elderly people usually suffer from multiple diseases, such as hypertension, diabetes and cardiovascular diseases, which are involved in the development of gout [26], emphasizing that early identification and timely prevention are important for elderly at high risk of gout. Additionally, the sex disparity is evident in the fact that the incidence and prevalence rates of gout is more than twice as high in men than women, which may be attributed to estrogen and progesterone promoting uric acid excretion in women [27,28], as well as men’s greater exposure to risk factors such as smoking, alcohol consumption and obesity [29,30]. The mentioned lifestyle-related risk factors were preliminarily validated in our study, especially excess adiposity being a foremost risk factor for gout. Adiposity increases serum urate levels and gout risk by reducing urate excretion and increasing urate production [31]. Ancillary analysis of a randomized dietary intervention trial showed healthy weight loss diets could reduce serum urate levels, especially among those with baseline hyperuricemia [32], whereas bariatric surgery has been associated with reductions in serum urate levels and incidence of gout and hyperuricemia [33]. Admittedly, the potential influencing factors analyzed in this study are limited, and future work is warranted to investigate other factors such as comorbidities, whose complex association with gout has received increasing attention [34].

Regarding the secular trends of gout burden, our study found that the total incidence and prevalence of gout in Ningbo Yinzhou area demonstrated an increasing trend over the past 11 years, although there was no significant change in the trend of female incidence. Similarly, increased incidence of gout has been reported previously in many countries over recent decades, and that the ageing populations in countries may further exacerbate these observed increases in incidence [34]. Notably, the burden of gout in Yinzhou district has risen markedly after 2018. Other possible explanations in the context of aging populations are, on the one hand, that changes in reimbursement systems over time may affect the frequency of diagnoses given to our study used registry data as drugs for the treatment of gout and hyperuricemia are gradually being included in the National Health Insurance Catalogue. For example, febuxostat, a potent non-purine xanthine oxidase inhibitor and generally considered a second-line urate-lowering therapy [1], was included in Class B of the Chinese National Health Insurance Catalog in 2019 [35], which greatly reduced the medication burden of patients with gout. On the other hand, the diagnostic criteria for gout in the guideline for the diagnosis and management of hyperuricemia and gout in China (2019) was based on the 2016 American Rheumatism Association/European Alliance of Associations in Rheumatology, and proposed the concept of subclinical gout for the first time, that is, patients with asymptomatic hyperuricemia who are found to have sodium urate crystal deposits and/or gouty bone erosions on imaging. Thus, the new classification criteria improved the early diagnosis of gout and identified more cases. Additionally, the increased incidence may also be attributed to the improvements in local diagnostic level, the establishment of new rheumatology and immunology clinics in some hospitals, and the increased awareness of patients to seeking medical care. While there have been improvements in areas such as diagnostic capacity and medical services for gout, these may not be sufficient to fully explain the markedly increased incidence of gout since 2018, thus, more evidence is still required to support our findings.

Our study utilizes a large, real-world regional health information platform to estimate the incidence and prevalence of gout in urban China over an extended period. This further provides epidemiological evidence on temporal trends as well as age- and gender-specific patterns of gout in a rapidly urbanizing population. The major strengths of this study include its population-based design, the large sample size of more than one million adults, and the comprehensive use of routinely collected electronic health records, which reduced recall bias and enabled robust analysis across subgroups. These findings highlight the growing need to strengthen primary care services and integrate gout management into chronic disease prevention programs from a public health perspective. Targeted interventions are particularly important for high-risk groups, such as elderly men, individuals with obesity, and those with lower educational attainment. Nevertheless, several limitations of the study still deserve attention. Firstly, misclassification (such as chondrocalcinosis) or underdiagnosis bias caused by the lack of validation of gout cases in our study was unavoidable, but the sensitivity analyses validated the robustness of the results. Secondly, the present study analyzed only limited factors. The impacts of extra factors on the incidence or prevalence of gout need also be considered, such as food intake, physical activity, use of urate-lowering drugs, and accessibility to health care. Finally, the study population was the resident population aged 18 years and above in Yinzhou, and the findings remain to be validated when extrapolated to the whole country.

## Conclusion

The incidence and prevalence rates of gout in Yinzhou District during 2011–2020 presented an increasing trend over time, which may be attributed to the improvement of diagnostic ability and the increase of healthcare utilization. Medical institutions and health authorities need to focus on the burden of gout and advocate adherence to favorable individualized lifestyles. In the future, epidemiological studies with larger scale and longer follow-up time are needed to supplement the gout data in China and to provide a reliable reference for the prevention and treatment of gout.

## Supplementary Material

Supplementary material.docx

## Data Availability

The datasets generated and/or analyzed during the current study are not publicly available but are available from the corresponding author on reasonable request.

## Abbreviations

AAPCs: Average annual percent changes
ASIR: Age-standardized incidence rates
ASPR: Age-standardized prevalence rates
BMI: Body mass index
CIs: Confidence intervals
SD: Standard deviation
YRHIP: Yinzhou Regional Health Information Platform
