# Supplementary material for: Incidence and prevalence of gout in Eastern China from 2011 to 2021: a retrospective population-based study
Source: Ann Med. 2025 Sep 19;57(1):2561230. doi: 10.1080/07853890.2025.2561230 (PMC12451967; doi:10.1080/07853890.2025.2561230)
Supplement: Supplementary material.docx [file IANN_A_2561230_SM0347.docx]

**Table S1. Keywords used to search for gout cases**

| **Diagnostic name** | **Chinese characters** | **Corresponding English translation** | **ICD-10** |
| --- | --- | --- | --- |
| Gout | 痛风 | Gout | M10 |
| Gout | 特发性痛风 | Idiopathic gout | M10.000 |
| Gout | 原发性痛风 | Primary gout | M10.000x094 |
| Gout | 痛风性关节炎 | Gouty arthritis | M10.002 |
| Gout | 痛风性滑囊炎 | Gouty bursitis | M10.003 |
| Gout | 心脏尿酸盐痛风石 | Gouty tophi of heart‌ | M10.004 |
| Gout | 痛风性肾结石 | Gouty kidney stones | M10.005 |
| Gout | 指关节痛风结节 | Gouty tophi of the finger joints | M10.006 |
| Gout | 铅性痛风 | Saturnine gout | M10.100 |
| Gout | 药物性痛风 | Drug-induced gout | M10.200 |
| Gout | 肾功能损害引起的痛风 | Gout secondary to renal impairment | M10.300 |
| Gout | 其他的继发性痛风 | Other forms of secondary gout | M10.400 |
| Gout | 痛风石 | Tophi | M10.900x093 |
| Gout | 痛风体质 | Gout diathesis | M10.901 |
| Gout | 耳痛风石 | Auricular tophus | M10.902 |
| Gout | 痛风结节 | Gouty nodule | M10.903 |
| Gout | 秋水仙碱 | Glucocorticoids | - |
| Gout | 别嘌醇 | Allopurinol | - |
| Gout | 非布司他 | Febuxostat | - |
| Gout | 丙磺舒 | Probenecid | - |
| Gout | 聚乙二醇化尿酸氧化酶 | Pegloticase | - |
| Gout | 苯溴马隆 | Benzbromarone | - |

**Table S2. Annual age-standardized incidence (/100,000 person-years, 95% CI) of early-onset gout by sex**

| **Year** | **Total** |  | **Male** |  | **Female** |
| --- | --- | --- | --- | --- | --- |
| 2011 | 62.69 (49.51-75.87) |  | 87.84 (65.04-110.65) |  | 41.09 (26.37-55.82) |
| 2012 | 83.73 (70.65-96.81) |  | 116.81 (94.24-139.39) |  | 54.95 (40.38-69.51) |
| 2013 | 52.50 (44.01-61.00) |  | 78.17 (62.99-93.35) |  | 30.09 (21.28-38.90) |
| 2014 | 50.79 (43.08-58.49) |  | 81.86 (67.59-96.13) |  | 23.49 (16.27-30.70) |
| 2015 | 40.17 (33.45-46.89) |  | 60.77 (48.60-72.93) |  | 22.24 (15.48-28.99) |
| 2016 | 50.16 (42.04-58.27) |  | 90.62 (74.72-106.51) |  | 14.44 (8.43-20.46) |
| 2017 | 36.69 (29.19-44.20) |  | 65.74 (51.19-80.29) |  | 10.78 (5.05-16.51) |
| 2018 | 40.03 (31.23-48.83) |  | 67.32 (50.62-84.02) |  | 15.86 (8.38-23.34) |
| 2019 | 85.08 (71.37-98.78) |  | 165.45 (137.74-193.15) |  | 15.92 (6.64-25.20) |
| 2020 | 101.50 (85.85-117.15) |  | 198.52 (165.13-231.91) |  | 21.35 (11.93-30.77) |
| 2021 | 162.70 (137.93-187.46) |  | 354.16 (293.98-414.35) |  | 17.28 (8.21-26.36) |
